# Differences in Brain Transcriptomes of Closely Related Baikal Coregonid Species

**DOI:** 10.1155/2014/857329

**Published:** 2014-01-29

**Authors:** Oksana S. Bychenko, Lyubov V. Sukhanova, Tatyana L. Azhikina, Timofey A. Skvortsov, Tuyana V. Belomestnykh, Eugene D. Sverdlov

**Affiliations:** ^1^M.M. Shemyakin and Yu.A. Ovchinnikov Institute of Bioorganic Chemistry, Russian Academy of Sciences, Miklukho-Maklaya Street, 16/10, V-437, 117997 Moscow, Russia; ^2^Limnological Institute, Russian Academy of Sciences, Ulan-Batorskaya 3, 664033 Irkutsk, Russia

## Abstract

The aim of this work was to get deeper insight into genetic factors involved in the adaptive divergence of closely related species, specifically two representatives of Baikal coregonids—Baikal whitefish (*Coregonus baicalensis* Dybowski) and Baikal omul (*Coregonus migratorius* Georgi)—that diverged from a common ancestor as recently as 10–20 thousand years ago. Using the Serial Analysis of Gene Expression method, we obtained libraries of short representative cDNA sequences (tags) from the brains of Baikal whitefish and omul. A comparative analysis of the libraries revealed quantitative differences among ~4% tags of the fishes under study. Based on the similarity of these tags with cDNA of known organisms, we identified candidate genes taking part in adaptive divergence. The most important candidate genes related to the adaptation of Baikal whitefish and Baikal omul, identified in this work, belong to the genes of cell metabolism, nervous and immune systems, protein synthesis, and regulatory genes as well as to DTSsa4 Tc1-like transposons which are widespread among fishes.

## 1. Introduction

Adaptation to different environmental conditions through filling available ecological niches may lead to population divergence and subsequently to the emergence of new species. Recently diverged populations are appropriate to study the genetic basis of primary evolutionary changes [1].

Having the unusual propensity for rapid speciation and adaptive radiation, Coregonidaeare becoming a model system for studying the genomic basis of adaptive divergence and reproductive isolation [2, 3]. Of particular relevance is the occurrence of both in North America (*Coregonus clupeaformis *complex) and Eurasia (*Coregonus lavaretus *complex) of lacustrine forms of whitefish that live in sympatry [3]. Phylogeographic studies confirmed the young age of these sympatric forms that evolved postglacially, less than 15,000 yr BP ([4, 5] and reviewed in [3]). For example, the limnetic dwarf ecotype of lake whitefish, *Coregonus clupeaformis, *derived in from the ancestral benthic normal ecotype evolved in parallel and independently in several postglacial lakes [6, 7]. Moreover, recent extensive gene expression studies have shown that, for some genes, this parallel phenotypic evolution of whitefish morphs is accompanied by parallelism in expression of the genes potentially underlying phenotypic divergence [1, 2, 8].

The subjects of our investigation were Baikal (lacustrian) whitefish *Coregonus baicalensis *Dybowski (bathypelagic bentophage) and Baikal omul* C. migratorius *Georgi (pelagic planctophage). The genomic sequences of the two organisms are still unavailable, thus excluding direct nucleotide-by-nucleotide comparison. However, analyses of Palearctic and Nearctic whitefishes [9, 10] as well as genealogical reconstruction of Baikal whitefishes [11, 12] showed that Baikal whitefish and omul are sister taxa and that they diverged from a common ancestor just 10–20 thousand years ago, probably due to the occupation of different trophic niches.

Baikal whitefish and omul arose after the last cool period (Sartanian glaciation, 34–10 thousand years ago) and represent one more case of sympatric postglacial whitefish divergence into pelagic and benthic niches [12]. However, their ancestor has diverged from all other coregonid fishes, including Baikal lacustrine-riverine whitefish, *Corgonus pidschian*, about 1.5 million years ago, when simultaneous speciation (or fast cladogenesis) took place in the south of East Siberia [13, 14]. This event gave rise to the several main clades of true whitefishes, among them the *C. clupeaformis *complex, the *C. lavaretus* complex, and all Lake Baikal coregonid fishes. The Baikal whitefish/omul pair is the only representative of its own clade, one of the main clades in the cluster of true whitefishes. The origin of the Baikal whitefish and omul ancestral form is likely Lake Baikal itself. This means that Baikal whitefish and the omul ancestral form inhabited Lake Baikal at least from the time of the formation of the true whitefishes group, moreover probably before the appearance of the genus *Coregonus *[14]. From at least the beginning of the Pleistocene, Lake Baikal has become not only large and deep but also oligotrophic and oxygenated water body inhabitable for higher animals [15]. It was hypothesized that isolation of pelagic and benthic forms within Lake Baikal was caused many times by Pleistocene climatic oscillations during 1.5 million years. Thus, Lake Baikal is the only place where true whitefish sympatric ecological divergence has been replicated many times within the same water body over such a long period of time. Another peculiarity consists in the multilevel pattern of intraspecific phenotypic divergence, especially pronounced in the pelagic form. Such multilevel structure is determined by availability of multiple ecological niches in the large, deep oligotrophic lake with a highly structured water body [13].

A whole genome comparison of Baikal whitefish and omul using subtractive hybridization did not reveal any difference between the two genomes [16]. All differential fragments found had a polymorphic character differed by single oligonucleotide substitutions or short (up to 35 nucleotides) insertions or deletions (indels). They belonged mostly to noncoding genomic regions: introns, and micro- and minisatellites as well as transposon-like structures but there was no divergence in protein coding genes detected. This result was additional evidence of the close relationship of Baikal whitefish and omul and might suggest that although genetic differences between coregonids at early stages of evolution are minimal in terms of genomic DNA sequence, they can still affect expression of some genes through, for example, mutations or heritable epigenetic modifications in *cis*-regulatory sequences. Recent analyses of transcription in closely related organisms using microarray technology confirmed that changes in expression of metabolic and regulatory genes might have a much larger impact on morphological traits than changes in structural genes [17, 18]. These changes may be further followed by large-scale alterations in genomic DNA [19].

It has been repeatedly reported that variations in neuron transcriptomes directly or indirectly correlate with behavioural differences between organisms [20, 21]. Therefore, by comparing the sets of genes transcribed in the brain of recently diverged fishes, one can hope to reveal candidate genes involved in adaptive divergence of populations [1].

Using the SAGE method (Serial Analysis of Gene Expression) [22], we performed a comparative study of Baikal whitefish and omul brain transcriptomes and found quantitative differences between them. Most of these differences belonged to the genes of cell metabolism, nervous and immune systems, protein synthesis, and regulatory genes as well as to Tc1-like transposons of the DTSsa4 family, which are widespread among fishes. Some of the differences revealed here may contribute to the phenotypic divergence of the two populations. The presence of parallelisms in phenotypic adaptation of Lake Baikal and other whitefishes toward the use of the pelagic niche accompanied by parallelism in differential pattern of expression is discussed.

## 2. Methods

### 2.1. Plasmid DNA

Growth and transformation of *E. coli* cells, preparation of plasmid DNA, agarose gel electrophoresis, and other nucleic acid manipulations were performed according to standard protocols [23] or to the recommendations of the manufacturers. The cells for plasmid extraction were grown overnight at 37°C in 5 mL of Luria-Bertani medium supplemented with ampicillin (0.1 mg/mL). Plasmid DNA was isolated using a Wizard Plus Miniprep DNA Purification System (Promega) according to the manufacturer's recommendations. Clone inserts were sequenced with an Amersham Biosciences/Molecular Dynamics MegaBACE 4000 Capillary Sequencer.

### 2.2. Oligonucleotides

Oligonucleotides were synthesized using an ASM-102U DNA synthesizer (Biosset Ltd., Russia). The primers specific for differential sequences were designed using the Primer3 software (http://bioinfo.ut.ee/primer3-0.4.0/).

### 2.3. Sample Collection

Live immature adult specimens of each Baikal whitefish and omul were collected by gill nets in August 2005 in the Maloye More strait region of Lake Baikal. The fishes were assigned to the two species according to the main diagnostic characteristics (i.e., counting the gill raker number on the first left gill arch and evaluation of the mouth position). Baikal lacustrine whitefish from the Maloye More region is a benthophage that has a subterminal mouth and 25–33 gill rakers on the first gill arch [23]. Omul is a planktophage with a terminal mouth and 37–51 gill rakers [23]. Whitefish individuals of our sample had a typical subterminal mouth, and the number of gill rakers varied from 25 to 31 (28 on average). As for omul individuals, they had a typical terminal mouth, and the number of gill rakers varied from 40 to 49 (44 on average). Six adult individuals were randomly collected in each species. Mean fork length and body mass for Baikal omul were 27.5 cm (SD = 3.1 cm) and 235 g (SD = 21 g), respectively. Mean fork length and body mass for Baikal whitefish were 34.5 cm (SD = 2.9 cm) and 489 g (SD = 28.5 g). Fishes were euthanized with 0.001% clove oil. Brain tissue samples were frozen immediately in liquid nitrogen and stored at −80°C. One specimen of each species was used for SAGE. Five specimens of each species were used for real-time PCR estimation of whitefish and omul brain cDNA transcription levels.

### 2.4. RNA Isolation and cDNA Synthesis

Total RNA was isolated from each sample of brain tissues using an SV Total RNA Isolation System (Promega) according to the manufacturer's recommendations. All RNA samples were treated with DNaseI to remove residual DNA. cDNA synthesis was performed using random hexamer primers with (RT+) or without (RT−) addition of PowerScript II reverse transcriptase (Clontech). The hexamer primers (12 pmol) were annealed in 11 *μ*L of a mixture containing 2 *μ*g total RNA. The mixture was heated for 2 min at 70°C and then chilled on ice for 10 min. To synthesize cDNA, the RT+ and RT− reaction mixtures were incubated at 37°C for 10 min and then at 42°C for 120 min.

### 2.5. Serial Analysis of Gene Expression

SAGE [22] was performed with an I-SAGE Long kit (Invitrogen) according to the manufacturer's protocol, starting with 100 *μ*g of total RNA. First, RNA samples were isolated from brain tissues of whitefish and omul (one sample for each species). The isolated RNA was incubated with magnetic beads (Dynabeads) and used to synthesize double-stranded cDNA according to a standard protocol. The cDNAs obtained were treated with NlaIII (NEB) restriction enzyme, and 3′ cDNA fragments were separated from other restriction fragments. The selected fragments were divided into two parts and ligated to two different adapters, each containing a MmeI restriction site. The ligated fragments were then treated with MmeI restrictase (NEB) to produce tags; the two mixtures were pooled together, and the tags were ligated to each other to give ditags. The ditags were PCR amplified, adapters were removed by digestion with NlaIII and separated from ditags in a 12% polyacrylamide gel, and the purified ditags were ligated to form linear concatemers.

The obtained concatemers, containing abundant representative cDNA fragments, were ligated into the pZErO-1 vector (Invitrogen) and cloned into *E. coli* cells. Independent recombinant clones were used to generate clone libraries arrayed in 96-well plates. Clones for sequencing were selected based on PCR screening.

### 2.6. Identification of cDNAs Corresponding to Tags Found by SAGE

To find the cDNA of a representative sequence (tag) from the SAGE libraries, double-stranded cDNA of the whitefish and omul brain (of the same two individuals that were used for SAGE, 3 *μ*g each) was digested overnight with PvuII restriction enzyme (100 U, Fermentas) in a volume of 300 *μ*L. The restricted cDNA was purified using a QIAquick PCR Purification Kit (Qiagen) and then ligated to the oligonucleotide adapters T7NotRsa (Table 1A) obtained by annealing the corresponding oligonucleotide and a short template (800 pmol each) in TM buffer (10 mmol L^−1^ Tris-HCl, pH 7.8, 10 mmol L^−1^ MgCl_2_). The ligation was performed overnight at 16°C using T4 phage DNA ligase (Promega). Then, two rounds of PCR were performed using the external T7 (round 1) and internal NotRsa (round 2) adapter primers (Table 1A), with the second primer being a tag oligonucleotide selected from the SAGE library. The PCR products were cloned and sequenced, and the sequences obtained were further used to design primers for real-time PCR.

### 2.7. Real-Time PCR

Real-time PCR was performed on all specimens of both species using an EVA green RT-PCR kit (Sintol, Russia) with 10 ng of the total cDNA samples as templates. Primers (Table 1B) were used at final concentrations of 0.2 *μ*mol L^−1^ each. The reactions were performed in a 25 *μ*L volume as follows: preincubation at 95°C (10 min) and then 40 cycles of 95°C for 30 s, 63°C for 30 s, and 72°C for 40 s. All real-time experiments were repeated in triplicate. The expression level of each gene was normalized using *GAPDH* as a reference gene. *GAPDH* oligonucleotide primers were designed against cDNA sequences annotated in GenBank records. At the end of the amplification, a dissociation curve was plotted to confirm the specificity of the product. To exclude contamination by genomic DNA, RT-experiments were done in parallel. The amplification efficiency of each primer set was determined using LinRegPCR [24]. The results were processed using the lin_reg_psr.exe and REST 2005 software [25].

### 2.8. Computer and Statistical Analysis

The libraries obtained for whitefish and omul were analysed using RIDGES software [26] as well as BlastAll and FormatDB programs from the BLAST software (NCBI, USA) [27]. RIDGES was used to extract tags from concatamer sequencing data and to calculate their amounts in libraries. 250 clones from each SAGE library were sequenced; 1894 and 2670 tags were identified in the whitefish and omul libraries, respectively (see Web Supplementary File Appendix 1) (Supplementary Material available online at http://dx.doi.org/10.1155/2014/857329). As the sequencing depth was not high enough to cover all transcripts, only the tags originating from the genes with the highest levels of expression were discovered in this way.

We found differentially expressed tags using online-based versions (Statistical Analysis of Transcript profiles, http://www.igs.cnrs-mrs.fr/spip.php?article168&lang=fr, and IDEG6, http://telethon.bio.unipd.it/bioinfo/IDEG6_form/) of the statistical test developed by Audic and Claverie [28]. This test gives the conditional probability of observing *y* number of tags in library *B*, given that *x* tags have been observed in libraries *A*, if *N*_*A*_ and *N*_*B*_ are the total number of tags for, respectively, library *A* and *B*, under the assumption that the null hypothesis is true and the null hypothesis is that the tag is expressed equally in both the conditions *A* and *B*. This method has been successfully used in several experiments to analyse data sets obtained by SAGE and RNA-Seq methods.

Due to the insufficient depth of sequencing, only 36 tags (see Web Supplementary File Appendix 2) were found to be differentially expressed between whitefish and omul, while the differences in the numbers of another 2732 unique tags were too small to be statistically significant. This fact also prevented our attempts to perform multiple testing corrections. Therefore, in order to confirm the obtained results, eight of the 36 differentially expressed genes were selected for qPCR instead of performing multiple testing corrections.

## 3. Results 

### 3.1. Baikal Whitefish and Omul Brain SAGE Libraries

The transcriptomes of the Baikal coregonids were compared by the SAGE method [22], an experimental technique designed to gain a direct and quantitative measure of gene expression. The SAGE method is based on the isolation of unique sequence tags (21 bp in length) of cDNAs followed by their concatenation serially and sequencing of the resulting long DNA molecules. It can give not only nucleotide sequences of many short fragments at a time and qualitative composition of transcriptomes, but also quantitative correlation between transcriptome components.

We obtained and characterized two libraries of short representative cDNA sequences (tags) from the brains of Baikal whitefish and omul (one library for one individual of each species). 250 clones from each library were sequenced that gave 1894 and 2670 tags for whitefish and omul, respectively (listed in Web Supplementary File Appendices 1 and 2).

A comparative quantitative characteristic of the whitefish tag library is shown in Table 2. The data obtained showed that only 3.9% of tags fall within the range of significance. The sequences of these “significant” cDNA fragments were compared to cDNA sequences of different organisms annotated in available databases. The results for tags with 70% to 100% identity level to known genes are presented in the Web Supplementary File Appendix 3. The table shows that most of tags that are more represented in one of the two libraries (relative value between 2 and 3, predominantly whitefish tags) are similar to segments of protein synthesis (ribosomal proteins 40S, S5, S11 and 60S L7, L13a, L39, L15, etc.) and regulatory (*Sox9a2, Cdc23, tnfsf5ip1, GRB10, btf2p44, PRKA*, etc.) genes. In contrast, the tags with a relative value between 0.5 and 0.3 (predominantly omul tags) are mostly similar to segments of metabolism genes (ATP synthase, H+ transporting, mitochondrial F0 complex, subunit b, and isoform 1; carbonic anhydrase 5B, Cytochrome P450 (CYP94C7); Na/K ATPase, alpha subunit, isoform 1c, etc.). However, a number of tags corresponding to cDNAs of metabolism genes were better represented in whitefish, while cDNA of 60S ribosomal protein L31 (clone ssal-rgh-513-300, *Salmo salar*) was similar to tags prevailing in omul.

Selected genes of the nervous and immune systems are more frequent in the whitefish library and others in the omul library (see Web Supplementary File Appendix 3). A number of tags more frequent in the whitefish library are similar to DTSsa4 Tc1-like DNA transposons.

For several of these apparently significant tags, we also determined sequences of the corresponding cDNAs and then confirmed the differences' reliability by real-time PCR.

### 3.2. Sequencing of cDNAs Corresponding to Tags

**Figure 1:**
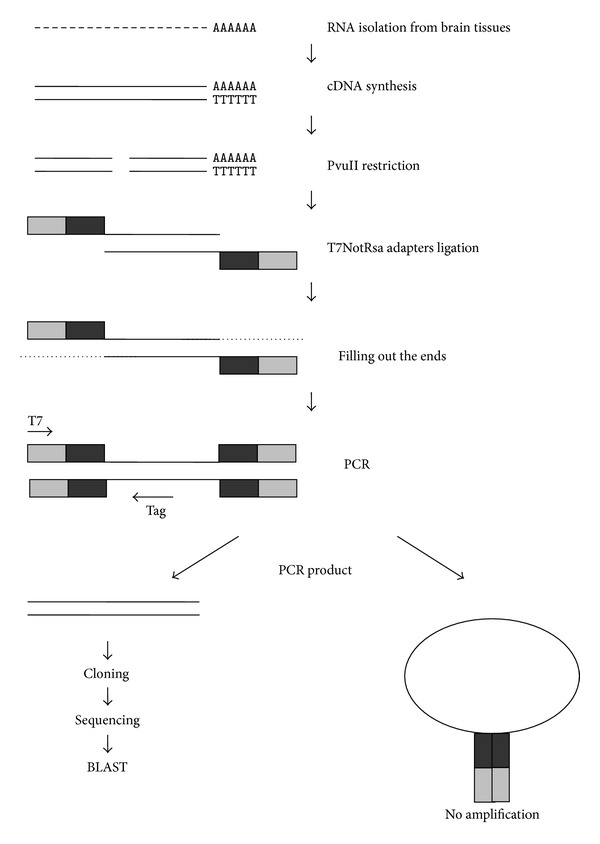
Identification of cDNAs corresponding to selected tags. cDNA synthesis was performed using the oligo(dT) primer. Double-stranded cDNA was hydrolyzed with PvuII followed by ligation to T7NotRsa suppression adapters (grey boxes). PCR primers are designated by arrows. “Tag” denotes an oligonucleotide primer designed according to the tag sequence found by SAGE.

Available databases contain no data on Baikal whitefish and omul cDNA sequences, so we selected some whitefish tags to partially sequence the corresponding cDNAs (the experiment schematic is shown in Figure 1). The obtained nucleotide sequences were compared to known cDNAs of different organisms. They were found to be highly (70–90%) similar to cDNA segments of such genes as fibroblast growth factor 12 (*Danio rerio*), ependymin-related protein 1 (*Danio rerio*), Na/K ATPase alpha subunit isoform 1c mRNA (*Oncorhynchus mykiss*), and other genes (Table 3).

### 3.3. Real-Time PCR Estimation of Whitefish and Omul Brain cDNA Transcription Levels

Real-time PCR of each cDNA fragment was performed with unique oligonucleotide primers. cDNA of those whitefish and omul individuals for which SAGE was carried out was used as a template. The PCR results confirmed that the expression of genes of the nervous (similar to netrin-G1 ligand, ependymin-related protein 1) and immune (tumour necrosis factor receptor superfamily, member 9) system as well as of the regulatory fibroblast growth factor 12 gene was two- to threefold enhanced in the brain of whitefish compared with that of omul.

The expression levels were also confirmed for the metabolism genes of NADH dehydrogenase subunit 5 and Na/K ATPase (higher in the whitefish brain) and alpha subunit isoform 1c (higher in omul brain).

To prove the identities of the amplified cDNA fragments, the products of real-time PCR were ligated into a vector, cloned, and sequenced (10 clones for each fish). For each analysed cDNA, the sequences obtained were at least 99% similar to each other. The differences were only in single clones and rare single-nucleotide substitutions did not affect the reading frames. This might be due to either DNA polymerase errors during amplification or to allelic variants of identical genes.

**Figure 2:**
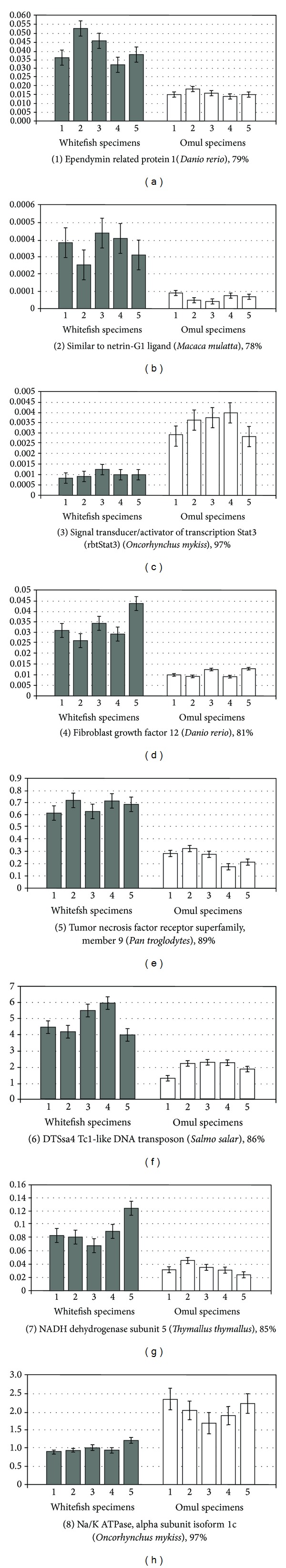
Transcription levels of genes in the whitefish and omul brain estimated by real-time PCR. %: similarity of the PCR product to mRNA of known genes. The *y*-axis shows the number of transcripts normalized to the content of the *GAPDH* housekeeping gene. Data represent mean ± S.E. of six independent experiments.

To exclude the possibility that the differences revealed resulted from individual polymorphisms in the expression level, we performed the real-time PCR analysis of the expression levels of the genes using cDNAs prepared from five individuals of each species (Figure 2).

## 4. Discussion

Data on genomic sequence and transcriptome comparison for the two important inhabitants of the Lake Baikal are currently unavailable. Earlier, we performed a whole-genome comparison using subtractive hybridization but were unable to detect any species-specific differences between Baikal whitefish and omul within the accuracy of the technique [16]. This result confirmed the close similarity of the two genomes, in accordance with the very recent divergence of the two whitefish species. However, it did not exclude the existence of undetected minor genetic or epigenetic differences differently affecting the level of gene expression.

The Baikal whitefish and omul pair represents one more case of multiple divergent true whitefish morphs, which evolved within lakes postglacially, less than 15,000 yr BP [5]. In all studied North American and Eurasian lakes, such sympatric morphs differ in the number of gill rakers, a highly heritable trait related to trophic utilization. Individual growth rate, age and size at maturity, diet, and habitat use also differ between morphs within lakes, but are remarkably similar across lakes within the same morph. Most of sympatric morphs are genetically different, and similar morphs from different lakes likely have a polyphyletic origin. These results are most compatible with the process of parallel evolution through recurrent postglacial divergence into pelagic and benthic niches in each of these lakes [3]. Moreover, during the last few years, gene expression studies have shown, for some genes, that this parallel phenotypic evolution of whitefish morphs accompanied by parallelism in expression of the genes potentially underlies phenotypic divergence [1, 2, 8, 29, 30].

The life history and ecology of the Lake Baikal sympatric pair have also been well documented [13, 31]. A similar pattern of phenotypic character displacement has contributed to the evolution of the Lake Baikal pelagic and benthic ecotypes. Is the presence of parallelism in phenotypic adaptation of Lake Baikal and other whitefishes toward the use of the pelagic niche accompanied by any parallelism in the differential pattern of expression? We used the SAGE approach to reveal the difference in brain gene expression levels between Baikal whitefish and omul and identified some of candidate genes involved in the divergence of these species.

### 4.1. Differential Expression in Brain of Baikal Whitefish and Omul

At least ∼4% of cDNA tags revealed quantitative differences between the fishes under study. Differential expression in the brains of closely related salmonids was also reported by other authors. For example, Aubin-Horth et al. found that 15% of transcripts were differentially expressed in the brain of salmonids (Atlantic salmon) with alternative developmental paths [32]. A differential expression was reported also for 10.5% of transcripts in the brain of salmonids (Atlantic salmon) living in natural conditions and those kept in captivity [33]. Finally, 11% of brain transcripts were differentially expressed in benthic and pelagic forms of whitefishes inhabiting lakes in the northeast of North America and diverged from a common ancestor. Candidate genes involved in the species divergence of these fishes belonged to functional categories of energy metabolism, protein synthesis, neural development, and, in some cases, regulatory genes, for example, a zinc-finger protein gene [1].

We show here that most tags that were two- to threefold more represented in whitefish than in omul were similar to protein synthesis and regulatory genes. In contrast, tags that were two- to threefold more represented in omul were mostly similar to segments of metabolism genes. Some tags differentially expressed in brain of whitefish and omul were similar to cDNA of genes of the nervous and immune systems and of DTSsa4 Tc1-like DNA transposons.

A considerable difference in expression was displayed by genes of the fish nervous system. An increased transcription level in whitefish was characteristic of two genes: ependimin related protein 1 (ERP) and a gene resembling the gene of netrin-G1 ligand (NGL-1). ERP is present at a high concentration in the cerebrospinal fluid of bony fishes, and it is associated with neuroplasticity, regeneration, and learning processes [34]. NGL-1 is mostly located in the cerebral cortex. Surface-bound NGL-1 stimulates the growth of embryonic thalamic axons, but free in solution the protein suppresses the growth [35]. In the brain of omul, an enhanced transcription level was observed for the *Stat3* gene of a signal transducer/activator of transcription. STAT3 is a multifunctional transcription factor of the central and peripheral nervous systems [36] and is necessary for differentiation of glial cells [37].

The identified genes of the nervous system might directly or indirectly affect the behavioural mechanisms of fishes, and the differential expression of these genes might facilitate the adaptation of the fishes to changeable environmental conditions. Two more genes of the nervous system known from the literature—the genes of troponin and SPARC—are thought to take part in the adaptive divergence of a lake whitefish species (*Coregonus* sp.) pair, dwarf (limnetic) and normal (benthic) whitefish [1]. These genes might be directly related to behaviour. SPARC is involved in neural development through signalling that allows neurons to end developmental migrations [38]. SPARC has also been proposed to modulate synaptic functions and to be involved in higher cortical functions in adult mammalian brains [39], making this a candidate for depth-preference behaviour [1]. Troponin is associated with actin and tropomyosin in the actin scaffold of muscle tissue [40]. In neurons, these molecules are collectively associated with neural development and growth [32, 41], thus potentially providing a link between troponin and behavioural differences between species pairs [1]. Thereby, in the nervous system, troponin and SPARC genes are needed for the development and growth of neurons. The differential expression of these genes in whitefishes is also suggested to affect the behaviour of fish, for example, the choice of depth of habitation. Thus, one of the potential targets of natural selection leading to behavioural differences might be the modulation of the expression of genes involved in the development of the nervous system.

Another way of adapting to changing environmental conditions can be accomplished by changing the expression levels of metabolism genes. One such gene is the gene of Na/K ATPase whose expression in omul is two- to threefold higher than that in whitefish. In nervous cells, Na/K ATPase is important for establishing the electrochemical gradient necessary for electroexcitability [42]. The enzyme is composed of a catalytic *α*-subunit and a glycoprotein *β*-subunit, which are suggested to be involved in the transport and stabilization of the enzyme complexes in membranes. The expression level of the catalytic *α*-subunit in Baikal omul was found to be ∼twofold higher than that in whitefish. A possible explanation is that Baikal omul leads a more active life than whitefish, thus needing a faster neural conduction velocity.

Genes of the respiration chain, such as Cytochrome c oxidase, Cytochrome P450, and ATP synthase, were also differentially expressed in whitefish and omul. Moreover, the transcript ratios for different families or subunits of these proteins in whitefish and omul were not equal. For instance, the brain of whitefish contained a larger number of ATP synthase d subunit transcripts than that of omul, whereas the content of b and g subunit transcripts was higher in omul than in whitefish. This could also be due to metabolism and life features of benthic Baikal whitefish and pelagic omul.

A considerable portion of tags that were two- to threefold better represented in the whitefish than in the omul library had a resemblance to segments of regulatory genes, for example, the gene of fibroblast growth factor (FGF) that plays a key role in proliferation and differentiation of various cells and tissues. In this work, we analysed young, rapidly growing and immature fish individuals of approximately the same age (3-4- and 4-5-year-old whitefish and omul, resp.). Therefore, the observed differential expression of regulatory genes might be, first of all, due to different growth rates of whitefish and omul: whitefish weighs more because the absolute body mass increment of whitefish is higher than that of omul during practically all the life [31, 43]. The same explanation may also be valid for the tags similar to mRNA segments of the genes of 60S and 40S ribosomal proteins, such as S5, S11, L7, and L13. An exception is the tags similar to mRNA of 60S ribosomal protein L31.

The whitefish library of representative sequences also contains more tags similar to the gene of TNF receptor and to some genes of the immune system, for example, the gene of MHC H-2 class II histocompatibility antigen *γ* chain. TNF receptor plays an important role in regulation of a wide spectrum of physiological process including the immune response.

It should be noted that a comparison of the Baikal whitefish and omul genomes using subtractive hybridization revealed quite a few differences in noncoding regions of the immune system genes, such as *TCR, MHC,* and *IgA*. In addition, many of the differential fragments located close to coding regions of these genes were 65–85% similar to TC1-like transposons [16]. We show here that this family's transposons have differential expression in brain tissues of the fishes under study, with the corresponding tags being ∼threefold better represented in the whitefish library. In the genomes of *Salmo salar* and *Danio rerio*, the Tc1-like transposon cDNA fragments identified in our work were in some cases mapped close to important regulatory genes, such as the genes of growth hormone, steroidogenic acute regulatory protein (StAR), coiled-coil transcriptional coactivator b2, homeobox protein HoxC13bb, guanine nucleotide binding protein G(o), and retinoic acid receptor *γ* b.

### 4.2. Parallelism in Differential Patterns of Expression between Pelagic and Benthic Ecotypes across Whitefish Sympatric Pairs

Over the last few years, gene expression studies have been intensively used to investigate the molecular basis of adaptive divergence between whitefish ecotypes in North American (taxon *Coregonus clupeaformis*) lakes [2, 8, 44]. These microarray studies were conducted both in controlled (laboratory) and natural (two lakes) environments, involved three life stages (embryos, juveniles, and adults) and three tissues (white muscle, liver, and brain). The hundreds of genes that showed differential patterns of transcription between pelagic and benthic whitefish across the three tissues were classified into at least 30 different functional groups. Of particular interest are those functional groups that were overrepresented in terms of number of parallel genes showing differences between pelagic and benthic whitefish relative to the total number of genes that were expressed in both ecotypes for each functional group. Pelagic whitefish consistently showed significant overexpression of genes potentially associated with survival through enhanced activity (energy metabolism, muscle contraction, homeostasis, lipid metabolism, and detoxification) whereas genes associated with growth (protein synthesis, cell cycle, and cell growth) were generally upregulated in benthic relative to pelagic whitefish [3]. In general, these transcriptomic studies combined with physiological data [45] show that energy metabolism is the main biological function involved in the divergence between pelagic and benthic whitefish. There is mounting evidence that selection has been acting more strongly on pelagic than benthic whitefish [8, 44].

In general, SAGE of Baikal whitefish and omul brain transcriptomes revealed a similar pattern of gene expression. Even with a low absolute amount of sequenced tags, it is evident that tags more represented in omul (pelagic ecotype) were mostly similar to segments of metabolism genes. In contrast, tags more represented in Baikal whitefish (benthic ecotype) had a resemblance to protein synthesis and regulatory genes.

Obviously, in Lake Baikal, in comparison with North American lakes, selection has been acting even more strongly on pelagic ecotype. The following peculiarities of Lake Baikal whitefish pair testify to it: (1) complete reproductive isolation of ecotypes/species by spawning times (autumn/winter) and places (rivers/lake shoals); (2) multilevel pattern of intraspecific phenotypic divergence, pronounced in the pelagic Baikal omul [13].

Transcriptome sequencing [46] of pelagic and benthic whitefish ecotypes in North American (taxon *Coregonus clupeaformis*) lakes displays even more clear parallelisms with the results of our SAGE on Lake Baikal whitefish and omul. The most salient finding of this work was that 14 genes involved in energy metabolism (both mitochondrial and nuclear) showed pronounced allele frequency differences and were also identified in several previous gene expression studies as differentially expressed in parallel between pelagic and benthic whitefish [46]. They are seven mitochondrial genes (cytochrome C subunits 1, 2, and 3; NADH-dehydrogenases 1, 4, and 5; and cytochrome b) and seven nuclear genes (cytochrome b-c1 complex subunit 6, ATP synthase subunit d, malate dehydrogenase, glyceraldehyde-3-phosphate dehydrogenase, creatine kinase, succinyl-CoA ligase, and angiopoietin-related protein 3 precursor). Special attention is given to genes of metabolic genes associated with the mitochondrion machinery. In our study, genes of the respiration chain were also differentially expressed in the Baikalian sympatric pair. Namely, the transcript ratios for different families or subunits of such proteins as Cytochrome c oxidase, Cytochrome P450, and ATP synthase in whitefish and omul were not equal. In general, as mentioned above, SAGE tags more represented in the Baikal omul (pelagic ecotype) were mostly similar to segments of metabolism genes.

“Nonmodel” species studied in their ecological context such as whitefish play an increasingly important role in ecological genomics [3]. Our work has confirmed that Lake Baikal is one more unique place to study genetic and phenotypic divergence among sympatric whitefish ecotypes. The comparative study of Baikal whitefish and omul brain transcriptomes revealed quantitative differences between species. Several genes involved with species diversity were identified and RT-PCR testified that differences in gene expression were not simple polymorphisms among fish within a species. Since the genomic sequence of both organisms studied remains unavailable, the generated data, albeit informative, remains speculative. Nevertheless, the presence of parallelism in differences of gene expression with similar sympatric whitefish pairs is evident. Thus, we hope this study will aid in future studies aimed at identifying the full genetic sequence of a number of expressed transcripts and will be a prelude to a more detailed analysis of adaptive variation and evolution of gene expression of Baikal whitefish and omul. The next step should be a comparative sequencing of transcriptomes. Undoubtedly, the questions that need to be further examined are the following. (1) Is parallelism in phenotypic adaptation of Lake Baikal and other whitefishes toward the use of the pelagic niche accompanied by parallelism in candidate gene transcription? (2) Is complete isolation between Lake Baikal whitefish ecotypes and multilevel pattern of intraspecific phenotypic divergence in pelagic ecotype accompanied by the extent of candidate gene transcription?

## Supplementary Material

Pairwise Audic and Claverie test are presented. RNA samples were used for cDNA synthesis and production of 21 nt tags. These tags were ligated to form ditags, and then concatemerized and cloned. 250 clones from each SAGE library were sequenced. 1894 and 2670 tags were identified in the whitefish and omul libraries, respectively (see Web Supplementary File Appendix 1). As the sequencing depth was not high enough to cover all transcripts, only the tags originating from the genes with the highest levels of expression were discovered this way.
Table S1: (Web Supplementary File Appendix 1) Complete set of short representative cDNA sequences (tags) extracted from concatamer sequencing data of the SAGE libraries obtained for whitefish and omul brain using RIDGES software. 1894 and 2670 tags were identified in the whitefish and omul libraries, respectively. A total of 2768 of unique tags were extracted and used for identification of differentially expressed genes by IDEG6.
Table S2: (Web Supplementary File Appendix 2) Identification of differentially expressed genes by IDEG6. The pairwise Audic & Claverie test was applied. 36 of 2768 tags were found to be differentially expressed between whitefish and omul, while the differences in the numbers of another 2732 unique tags were too small to be statistically significant.
Table S3: (Web Supplementary File Appendix 3). Comparison of some cDNA fragments (tags) with cDNA sequences of different organisms, annotated in available databases. The results for tags with 70–100% identity level to known genes are presented. We considered as statistically significant differences between whitefish and omul libraries in the range 0.6 > Dif_wh_ >1.6 that corresponds to the statistical significance level p>0.999. Difwh is the relative content of a given tag in the whitefish library (relative to omul), Dif_wh_= (N_wh_ / N_om_) *х* 1.41, N_wh_ and N_om_ are the numbers of the given tag in the libraries of Baikal whitefish and omul, respectively, and 1.41 is the correction coefficient equal to the ratio 2670/1894.

## Figures and Tables

**Table:** (a)

Name of the oligonucleotides	Oligonucleotides sequence (5′-3′)	Annealing temperature (°C)
External primer T7	CTAATACGACTCACTATAGGGC	66
Internal primer NotRsa	AGCGTGGTCGCGGCCGAGGT	68
GAPDH for. GAPDH rev.	GTGGACGGCCCCTCTGC TCTGGTGGGCACCACGG	63
Suppression adapter (equimolar mixture of preliminary annealed oligonucleotides T7NotRsa and Rsa_10)	T7NotRsa CTAATACGACTCACTATAGGGCAGCGTGGTCGCGGCCGAGGT Rsa_10 ACCTGCCCGG	
Tag no. 89	GTACTTTAATGGATGATCTCC	56
Tag no. 81	GTACAAAAAGACTGCTGTTCC	60

**Table:** (b)

Name of the oligonucleotides	Oligonucleotides sequence (5′-3′)	Annealing temperature (°C)
GAPDH for. GAPDH rev.	GTGGACGGCCCCTCTGC TCTGGTGGGCACCACGG	63
1ep for. 1ep rev.	TGGTGTAGGTCTCCTGGAC CTCCACCTATGAGGACCAG	63
2net for. 2net rev.	CAGCTCCTCCAGACGCACC CGCTGGAGCTGTTCGACAAC	63
3st for. 3st rev.	TGGAGGAGAAGATAGTGGACTTGT AGTAATCTGACTTTGTTGGTGAACT	63
4fgf for. 4fgf rev.	CGGTACAGCGTCGATGAGTAG CACCATAAATGGGACCAAGG	63
5tnf for. 5tnf rev.	CATTGCAGTCCTAGTCTCTCT AGTGGTATCAACGCAGAGTAC	63
6tr for. 6tr rev.	CAGGACAATGACCCAACACAC TGAGGTCGGGGGATTGTG	63
7deh for. 7deh rev.	GAGGAGGTATTTAAAGAATCGG CCTCAGCTTTAAATTTGACCAC	63
8atp for. 8atp rev.	GGCAGGTGAACTCCACAATC CAGGAGTGCTGGAATCAAGG	63

**Table 2:** A comparative quantitative characteristic of the whitefish tag library.

Representation of a given tags in the whitefish library relative to omul (Dif_wh_)	Relative content in the whitefish library, %
2.7–3.7	2.0
1.7–2.6	1.2
0.6–1.6	96.1*
0.3–0.5	0.7
In total	3.9

*Tags which turned outside the range of significance. We considered as statistically significant differences between whitefish and omul libraries in the range 0.6 > Dif_wh_ > 1.6 that corresponds to the statistical significance level *P* > 0.999. Dif_wh_ is the relative content of a given tag in the whitefish library (relative to omul), Dif_wh_ = (*N*_wh_/*N*_om_) × 1.41, *N*_wh_ and *N*_om_ are the numbers of the given tag in the libraries of Baikal whitefish and omul, respectively, and 1.41 is the correction coefficient equal to the ratio 2670/1894.

**Table 3:** Transcription levels of genes in the whitefish and omul brain estimated by real-time PCR.

	Accession	Similarity to mRNA of known genes, %	Accession of mRNA of known genes	*Q*wf ± S.E.	*Q*omul ± S.E.	R ± S.E.
1	GenBank: GR918015	Ependymin related protein 1 (*Danio rerio*), 79	GenBank: NM_001002416.1	3.7 × 10^−2^ ± 0.4 × 10^−2^	1.5 × 10^−2^ ± 0.12 × 10^−2^	2.79 ± 0.6
2	GenBank: GR918016	Similar to netrin-G1 ligand (*Macaca mulatta*), 78	GenBank: XM_001090447.1	3.9 × 10^−4^ ± 0.9 × 10^−4^	0.89 × 10^−4^ ± 0.2 × 10^−4^	4.12 ± 0.8
3	GenBank: GR918017	Signal transducer/activator of transcription Stat3 (rbtStat3) mRNA (*Oncorhynchus mykiss*), 97	GenBank: OMU60333	8.2 × 10^−4^ ± 3.1 × 10^−4^	29.09 × 10^−4^ ± 5 × 10^−4^	0.33 ± 0.05
4	GenBank: GR918020	Fibroblast growth factor 12 (*Danio rerio*), 81	GenBank: BC124640.1	3.1 × 10^−2^ ± 0.42 × 10^−2^	1.05 × 10^−2^ ± 0.09 × 10^−4^	2.62 ± 0.6
5	GenBank: GR918021	Tumor necrosis factor receptor superfamily, member 9 (*Pan troglodytes*), 89	GenBank: XM_001157779.1	0.61 ± 0.04	0.27 ± 0.02	2.45 ± 0.6
6	GenBank: GR918018	DTSsa4 Tc1-like DNA transposon (*Salmo salar*), 86	GenBank: EF685957.1	4.43 ± 0.41	1.3 ± 0.18	3.48 ± 0.5
7	GenBank: GR918022	NADH dehydrogenase subunit 5 (*Thymallus thymallus*), 85	GenBank: AF270855	8.2 × 10^−2^ ± 0.9 × 10^−2^	3.5 × 10^−2^ ± 0.42 × 10^−2^	2.27 ± 0.24
8	GenBank: GR918019	Na/K ATPase alpha subunit isoform 1c mRNA (*Oncorhynchus mykiss*), 97	GenBank: AY319389.1	0.86 ± 0.05	2.38 ± 0.21	0.35 ± 0.03

Data represent mean ± S.E. (standard error) of six independent experiments.

*Q*: the number of transcripts normalized to the number of *GAPDH* gene transcripts.

R: relative expression~*Q*whitefish/*Q*omul.
