# Supplementary material for: Differences in Brain Transcriptomes of Closely Related Baikal Coregonid Species
Source: Biomed Res Int. 2014 Jan 29;2014:857329. doi: 10.1155/2014/857329 (PMC3956407; doi:10.1155/2014/857329)
Supplement: Supplementary file 1 — Pairwise Audic and Claverie test are presented. RNA samples were used for cDNA synthesis and production of 21 nt tags. These tags were ligated to form ditags, and then concatemerized and cloned. 250 clones from each SAGE library were sequenced. 1894 and 2670 tags were identified in the whitefish and omul libraries, respectively (see Web Supplementary File Appendix 1). As the sequencing depth was not high enough to cover all transcripts, only the tags originating from the genes with the highest levels of expression were discovered this way. Table S1: (Web Supplementary File Appendix 1) Complete set of short representative cDNA sequences (tags) extracted from concatamer sequencing data of the SAGE libraries obtained for whitefish and omul brain using RIDGES software. 1894 and 2670 tags were identified in the whitefish and omul libraries, respectively. A total of 2768 of unique tags were extracted and used for identification of differentially expressed genes by IDEG6. Table S2: (Web Supplementary File Appendix 2) Identification of differentially expressed genes by IDEG6. The pairwise Audic & Claverie test was applied. 36 of 2768 tags were found to be differentially expressed between whitefish and omul, while the differences in the numbers of another 2732 unique tags were too small to be statistically significant. Table S3: (Web Supplementary File Appendix 3). Comparison of some cDNA fragments (tags) with cDNA sequences of different organisms, annotated in available databases. The results for tags with 70–100% identity level to known genes are presented. We considered as statistically significant differences between whitefish and omul libraries in the range 0.6 > Difwh >1.6 that corresponds to the statistical significance level p>0.999. Difwh is the relative content of a given tag in the whitefish library (relative to omul), Difwh= (Nwh / Nom) х 1.41, Nwh and Nom are the numbers of the given tag in the libraries of Baikal whitefish and omul, respectively, and 1.41 is th [file 857329.f1.pdf]

Appendices

A. File Format: XLS

Library of Short Representative cDNA Sequences (Tags) from the Brain of Baikal Whitefish and Omul

Description

250 clones from the library were sequenced. 1894 and 2670 tags were identified in the whitefish and omul libraries.

B. File Format: PDF

Results of Running IDEG6

Description

Pairwise Audic and Claverie test are presented. RNA samples were used for cDNA synthesis and production of 21 nt tags. These tags were ligated to form ditags, and then concatemerized and cloned. 250 clones from each SAGE library were sequenced. 1894 and 2670 tags were identified in the whitefish and omul libraries, respectively (see Web Supplementary File Appendix 1). As the sequencing depth was not high enough to cover all transcripts, only the tags originating from the genes with the highest levels of expression were discovered this way.

We found differentially expressed tags using online-based versions (Statistical Analysis of Transcript profiles, http://www.igs.cnrs-mrs.fr/spip.php?article168&lang=fr and IDEG6, http://telethon.bio.unipd.it/bioinfo/IDEG6_form/) of the statistical test developed by Audic and Claverie (Audic and Claverie, 1997, Genome Research). This test is based on the assumption that the probability of observing n tags of the same gene is approximated by the Poisson distribution:

$$p(n) = \frac{e^{-\lambda}\lambda^{n}}{n!},$$

where $\lambda \geq 0$ is the actual number of tags of this type per N clones in the library. The statistic is calculated according to equation below:

$$p(y \mid x) = \left(\frac{N_B}{N_A}\right)^{y} \frac{(x+y)!}{x!\,y!\,(1 + N_B/N_A)^{(x+y+1)}}$$

The Audic and Claverie test gives the conditional probability of observing y number of tags in library B, given that x tags have been observed in library A, if N_A and N_B are the total number of tags for, respectively, library A and B, under the assumption that the null hypothesis is true and the null hypothesis is that the tag is expressed equally in both the conditions A and B. This method has been successfully used in several experiments to analyse data sets obtained by SAGE and RNA-Seq methods.

C. File Format: PDF

Comparison of Some cDNA Fragments (Tags) with cDNA Sequences of Different Organisms, Annotated in Available Databases

Description

The results for tags with 70–100% identity level to known genes are presented. We considered as statistically significant differences between whitefish and omul libraries in the range $0.6 > \text{Dif}_{\text{wh}} > 1.6$ that corresponds to the statistical significance level $P > 0.999$.

Dif_{wh} is the relative content of a given tag in the whitefish library (relative to omul), $\text{Dif}_{\text{wh}} = (N_{\text{wh}}/N_{\text{om}}) \times 1.41$, N_{wh} and N_{om} are the numbers of the given tag in the libraries of Baikal whitefish and omul, respectively, and 1.41 is the correction coefficient equal to the ratio 2670/1894.
